# Associations Between Self-Reported Injury History, Physical Complaints, and Medical Attention Injury During Army Basic Military Training

**DOI:** 10.1093/milmed/usaf185

**Published:** 2025-05-19

**Authors:** Neil Gibson, Jace R Drain, Matthew Whalan, Sean Williams, Herbert Groeller, John A Sampson

**Affiliations:** School of Medical, Indigenous and Health Sciences, Faculty of Science, Medicine and Health, University of Wollongong, Wollongong, NSW 2522, Australia; Land Division, Defence Science and Technology Group, Fishermans Bend, VIC 3207, Australia; School of Medical, Indigenous and Health Sciences, Faculty of Science, Medicine and Health, University of Wollongong, Wollongong, NSW 2522, Australia; Medical Department, Football Australia, Sydney, NSW 2000, Australia; Department for Health, University of Bath, Bath BA2 7AY, United Kingdom; School of Medical, Indigenous and Health Sciences, Faculty of Science, Medicine and Health, University of Wollongong, Wollongong, NSW 2522, Australia; School of Medical, Indigenous and Health Sciences, Faculty of Science, Medicine and Health, University of Wollongong, Wollongong, NSW 2522, Australia

## Abstract

**Introduction:**

Effective and easily implementable methods to reduce the incidence and burden of injury during Army basic military training (BMT) are desirable. This study therefore investigated (1) the association between prior injury history and medical attention (MA) injury and (2) the association and accuracy of daily self-reported physical complaints on the incidence of MA injury, during Army BMT.

**Materials and Methods:**

Recruits (*n *= 625, male = 524; female = 101; age: 22 ± 6 years [range: 17-55 years]) completed a 12-month prior injury history questionnaire during week 1 and throughout BMT reported physical complaints daily, using a modified Oslo Sports Trauma Research Centre Questionnaire on Health Problems (OSTRC-H). Medical attention injuries were recorded via physiotherapy reports. Cox proportional hazard regressions explored the association between prior injury and MA injury. Generalized linear mixed-effects models were used to model the association between OSTRC-H responses and an MA incident injury within 7 days. The predictive ability and accuracy of OSTRC-H responses were also assessed.

**Results:**

Prior injury was not significantly associated with a greater risk of MA injury during BMT. Self-reported physical complaints effecting “participation” (“Full participation, but with injury/illness”: OR = 2.23, 95% CI 1.97-2.52; “Reduced participation due to injury/illness”: OR = 3.19, 95% CI 2.54-4.00), “severity” (“To a mild extent”: OR = 2.19, 95% CI 1.91-2.51; “To a moderate extent”: OR = 2.83, 95% CI 2.38-3.36; “To a severe extent”: OR = 4.50, 95% CI 3.26-6.21), and “location” (OR = 2.19, 95% CI 1.96-2.45) were significantly associated with greater odds of MA incident injury within 7 days. Spine (OR = 4.39, 95% CI 3.07-6.30), upper extremity (OR = 2.45, 95% CI 1.76-3.40), and lower extremity (OR = 2.73, 95% CI 2.40-3.40) physical complaints were significantly associated with an MA incident injury to the corresponding general body region within 7 days. Using the presence of a physical complaint to indicate the occurrence of an MA incident injury within 7 days resulted in a high number of false positives and false negatives (area under the curve: 0.51-0.66).

**Conclusions:**

Independently, self-reported 12-month prior injury was not significantly associated with a greater risk of an MA injury during BMT. Daily self-reported physical complaints may however flag increased MA injury risk, which could help prevent more severe injuries.

## INTRODUCTION

Army basic military training (BMT) aims to provide foundational training to prepare recruits for military life.^1^ Yet, military training is not without risk and injuries can occur.^1,2^ Lower extremity musculoskeletal injuries are consistently reported as the most common during BMT, with overuse the most frequently reported injury mechanism.^2–5^ Injury during BMT can delay graduation and contribute to attrition, consequently increasing training costs and impacting upon the supply of personnel to the trained workforce.^6,7^ Effective and easily implementable methods to reduce the incidence and burden of injury during BMT are therefore desirable.^6,8^

Injury is multifactorial, complex, and dynamic.^9,10^ Intrinsic risk factors may predispose an individual to injury,^10^ with prior injury commonly reported as a risk factor for future injury within sport and military settings.^5,11–13^ Within recruit populations, several other intrinsic injury risk factors have also been identified including entry-level fitness^1,5^ and self-reported physical activity before training.^12^ However, risk factors that are assessed at a single time point (e.g., baseline or week 1) or that are nonmodifiable potentially fail to account for a recruit’s dynamic risk of injury during BMT, and continuous monitoring strategies may offer a more practical assessment of injury risk.^14^

During BMT, physical complaints can arise,^3,4^ reflecting the presence of perceived pain, and may progress to a more serious injury.^15^ Self-report data collection methods, such as the Oslo Sports Trauma Research Centre Questionnaire on Health Problems (OSTRC-H), can be used to capture physical complaints,^16,17^ and within sport, the risk of a time-loss injury (within 7 days) has been reported to be greater when preceded by a self-reported physical complaint.^18^ Recruits who drop out of BMT due to injury have also been observed to report more physical complaints and higher ratings of pain in comparison to recruits who graduate or discharge at their own request.^15^ As such, daily self-reported physical complaints may offer a simple method to identify recruits who may be at an increased risk of medical attention (MA) injury during BMT.

Therefore, the aims of this study were 2-fold: (1) to investigate the association between prior injury and MA injury during Army BMT and (2) to assess the association and accuracy of daily self-reported physical complaints on the incidence of MA injury during Army BMT.

## MATERIALS AND METHODS

### Participants and Study Design

A prospective cohort study including 625 recruits (male = 524; female = 101; age: 22 ± 6 years [range: 17-55 years]), from 16 platoons (8 intakes), undertaking BMT at the Army Recruit Training Centre, Kapooka (Australia), was conducted during 2019. All platoons commenced the standardized 12-week BMT course between July and September. Study procedures and risks were explained verbally and provided in writing to recruits during week 1 (day 2) before written informed consent was provided. Seventeen female recruits, who undertook the Army pre-conditioning program, provided consent upon joining a study platoon in week 3.

### Experimental Design

#### MA injury recording

Medical attention injuries were recorded by physiotherapists at the Army Recruit Training Centre using an “any physical complaint” injury definition.^19^ Following recruit consultation, physiotherapists recorded the date, body part affected, injury type, and mechanism (overuse or trauma) for all injuries presented using a standardized data-collection form. Forms were routinely collected and manually processed by a member of the research team and subsequently assigned an International Classification of Disease, tenth revision, Clinical Modification (ICD-10 CM) code (to 6 digits when available, minimum 4 digits), based on body part affected and injury type. An MA injury was defined as the first report of a specific injury (i.e., ICD-10 CM code) by a recruit to the physiotherapists, and in an attempt to avoid overestimating injuries, at least 30 days had to pass after an individual’s last report with a specific ICD-10 CM code before the same code could again be used to document a new “incident injury.”^2,20^ If no date was recorded on the standardized collection form the report was excluded from analyses, unless it was the only report to the specified body region for the recruit.

#### Twelve-month prior injury history data collection and preparation

Following the provision of consent, recruits completed a customized 12-month prior injury history questionnaire to document injuries that (1) required time off work or sporting pursuits, “time loss injury,” and (2) did not require time off work or sporting pursuits, “non-time loss injury.” Questionnaire responses were coded as 0 = no or 1 = yes, within 3 categories: did the recruit have a prior (1) “time loss injury”; (2) “non-time loss injury”; and (3) “any prior injury” (i.e., any report in category 1 or 2).

#### Daily self-reported physical complaint collection and preparation

Each evening (days 2-79, or until a recruit left the study [i.e., back squadded or attrition]), recruits indicated the presence and consequences of any physical complaint using a modified OSTRC-H,^16^ consisting of 5 questions: Q1 (participation) = “Have you had any difficulties partaking in military training/activities/education due to injury, illness or other health problems today?”; Q2 (severity) = “To what extent have you experienced symptoms/health complaints today?”; and Q3 (location), presented a body map with instruction to “select the circle that best describes the location of your injury” and for illness to check the option of “illness” only (no location). A “no injury/illness” check option was provided to ensure an answer was required for all questions. Questions 2 and 3 were repeated as question 4 and 5, allowing recruits to indicate their 2 “worst” health problems daily (**Supplementary Figure S1**, daily modified OSTRC-H). The OSTRC-H was presented within a booklet (daily dairy), containing daily questionnaires for the week (7 days). Booklets were collected and replaced each Monday. Daily self-reports were scanned (Canon DRC240, Canon, Tokyo, Japan) and subsequently processed using Remark Office OMR software (Remark, Malvern, PA, USA).

Daily self-reports were matched to recruit days in training. Medical attention incident injuries were aligned with the corresponding date to determine if a self-report was within 7 days of an incident injury.^18^ Daily self-reports with a checked body map location were classified as an injury report and aligned with body locations from the taxonomy.^20^ An accumulated injury score was not calculated,^16^ as a modified OSTRC-H was used, rather “participation” (question 1), “severity” (questions 2 and 4), and “location” responses were analyzed.^18^ For each day, the max severity reported was assessed, while location responses were coded as 0 = “no location” or 1 = “at least one body location.” Furthermore, for each general body region (i.e., head and neck, spine and back, torso, upper extremity, and lower extremity), daily responses were coded as 0 = “no body region complaint” or 1 = “at least one body region complaint.” For recruits who completed BMT, self-reports on day 79 were excluded from analyses as recruits had limited opportunity to seek MA on day 80 (march out). As an inability to participate in BMT could arise from a recruit seeking MA or prescribed training restrictions provided by medical personnel, self-reported responses of “could not participate” were considered indicative of a recruit having already sought MA and removed from the analyses. All “illness” complaints were excluded. If a recruit reported an “illness” and injury complaint on the same day, it could not be determined which impacted upon the reported effect on participation; therefore, the participation question was removed from the analyses. In the absence of a daily self-report the given day for that recruit was excluded, yet, if at least one question was answered (e.g., location), only missing responses were excluded (e.g., question 1 and 2). All missing data are reported for transparency.^21^

### Statistical Analyses

All statistical analyses were performed using “R” (version 4.0.2, R Foundation for Statistical Computing, Vienna, Austria). Due to recruit attrition, survival analyses (Cox proportional hazard regression) were performed to investigate the association between prior injury and time to MA injury, using the “survival” package.^22^ Time at risk was calculated as the number of days within a study platoon (e.g., BMT start date [day 0] to training completion date). If a recruit sustained an injury, their time at risk was calculated until that day, while if a recruit did not complete training (e.g., discharged), their time at risk was censored at the day they left a study platoon. In each model, prior injury represented the predictor variable (i.e., “time-loss injury,” “non-time loss injury,” or “any prior injury”). Model estimates were exponentiated, using the exponential function, to provide hazard ratios and 95% CIs.

Generalized linear mixed-effects models (binomial, link = logit) were used to model the association between self-reported OSTRC-H responses and the incidence of an MA incident injury within 7 days, using the “lme4” package.^23^ In each model, the dependent variable was the injury indicator (0 = “no incident injury within 7 days”; 1 = “incident injury within 7 days”). OSTRC-H responses were entered as the fixed effect predictor variable with recruit identity included as a random effect. The risk of an incident injury to the same general body location as the self-reported physical complaint was also assessed. Model parameter estimates were exponentiated to provide an OR and 95% CI. Marginal (variance explained by the fixed effect alone) and conditional (variance explained by both the fixed and random effects) *R*^2^ values are presented.^24^ The predictive ability of OSTRC-H responses was assessed using receiver operating characteristic (ROC) curves, which examines the discriminant ability of a marker to classify recruits in 2 groups and plots the true positive rate (sensitivity) against the true negative rate (specificity) producing an area under the curve (AUC). An AUC of 1 (100%) represents perfect discriminant power, while 0.5 (50%) represents no discriminatory power.^25,26^ The 95% CIs are reported based on bootstrap analysis with 2,000 iterations using the “pROC” package.^27^ An AUC of >0.7 and CIs of >0.5 were used as generic benchmarks for interpreting “acceptable” discriminant ability.^28^ To assess accuracy, questionnaire responses were used to create 2 × 2 contingency tables and calculate sensitivity, specificity, positive and negative likelihood ratios and predictive values, and overall accuracy. For all analyses, statistical significance was set at *P* < .05.

## RESULTS

### Details of Cohort Training Days and MA Injuries

For the 625 recruits, there were 45,218 (38,358 males and 6,860 females) individual recruit training days, with recruits averaging 72 days (minimum = 4, first quartile = 80, median = 80, third quartile = 80, maximum = 101) in BMT (**Supplementary Table S1**, training outcome for the recruits involved in the present study). During BMT, 226 recruits (36.2%) had ≥1 MA incident injury with a total of 596 incident injuries recorded (spine and back 52; lower extremity 457; upper extremity 67; torso 17; head and neck 1; 2 other [unspecified]) from 949 physiotherapy reports.

#### Twelve-month injury history

The response rate to the prior injury questionnaire was 98.2%. Accordingly, 614 recruits (male = 514; female = 100; age: 22 ± 6 years [range: 17-55 years]) were included in the prior injury analyses with 221 recruits (36%) sustaining ≥1 incident injury during BMT (spine and back 52; lower extremity 442; upper extremity 67; torso 15; head and neck 1; 2 other [unspecified]). For the 12 months before BMT, 223 recruits (36.3%) reported “any prior injury,” with 170 reporting a prior “non-time loss injury,” 16 reporting a prior “time-loss injury,” and 37 reporting both a prior “non-time loss injury” and “time-loss injury.” No significant association between “any prior injury” (hazard ratio = 1.31 [95% CI: 1.00-1.71], *P* = .051), prior “time-loss injury” (hazard ratio = 1.50 [95% CI: 0.98-2.30], *P* = .06), or prior “non-time loss injury” (hazard ratio = 1.28 [95% CI: 0.98-1.68], *P* = .08) and MA injury during BMT was observed.

Of the 223 recruits reporting “any prior injury,” 90 (40.4%) sustained an MA injury during BMT. In comparison, 131 (33.5%) of the 391 recruits reporting “no prior injury” sustained an MA injury during BMT. Eighty-three (40.1%) of the 207 recruits reporting a prior “non-time-loss injury” and 137 (33.7%) of the 407 recruits reporting no prior “non-time loss injury” sustained an MA injury during BMT. Twenty-four (45.3%) of the 53 recruits reporting a prior “time-loss injury” and 197 (35.1%) of the 561 recruits reporting no prior “time-loss injury” sustained an MA injury during BMT.

#### Daily self-reported physical complaints

**Figure 1.**
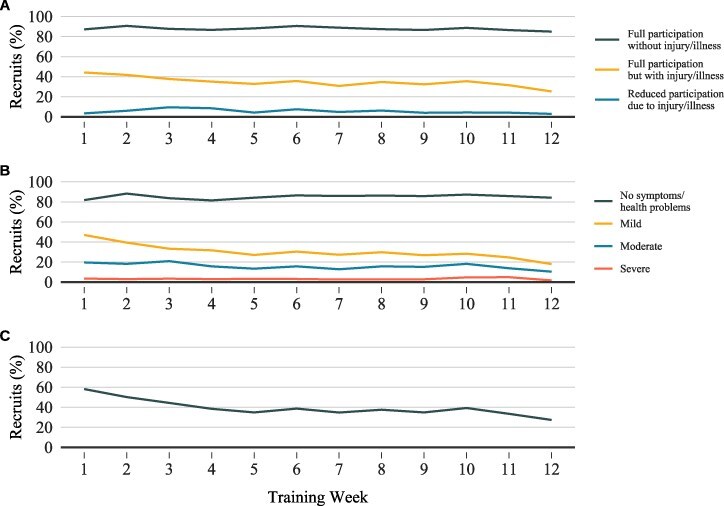
(A) The percentage of recruits who provided at least one report with the given response to the participation question each week. (B) The percentage of recruits who provided at least one report with the given response to the severity question each week. (C) The percentage of recruits who selected a body location for the location question each week.

No OSTRC-H responses were obtained for 7 recruits. Therefore, 618 recruits (male = 519; female = 99; age: 22 ± 6 years [range: 17-55 years]) were included in the daily self-report analyses, totaling 43,893 study (i.e., starting from day 2) training days. After excluding day 79 reports (*n* = 534), no responses (*n* = 3769) (compliance 91.3%), and reports of “could not participate” (*n* = 278) and “illness” (*n* = 2), 39,310 days were retained for analyses. For participation, 36,881 (2,371 injury and illness reports, 58 no response) days, consisting of 29,182 self-reports of “full participation without injury/illness,” 7066 “full participation, but with injury/illness,” and 633 “reduced participation due to injury/illness,” were included in the analyses. For severity, 36,400 days were analyzed (2,910 no responses). The max severity on 28,596 days was “no symptoms/health problems,” 5169 days “mild,” 2249 days “moderate,” and 386 days “severe.” A body location was checked on 8,443 of the 39,310 days with 519 head and neck, 1,212 spine and back, 334 torso, 1,677 upper extremity, and 5,876 lower extremity complaints reported. The percentage of recruits reporting each “participation,” “severity,” and a “location” response each week are presented in Figure 1. Due to the absence of a date on physiotherapy collection forms, 11 incident injuries (lower extremity 8, upper extremity 3), from a total of 9 recruits, were excluded. Therefore, 584 incident injuries (spine and back 52; lower extremity 448; upper extremity 64; torso 17; head and neck 1; 2 other [unspecified]) from 224 recruits with ≥1 incident injury were included in the OSTRC-H analyses.

Self-reported “participation,” “severity,” and “location” responses indicative of a physical complaint were each significantly associated with an MA incident injury within 7 days (**Table 1**). Spine, lower extremity, and upper extremity complaints were each independently associated with an MA incident injury to the corresponding body region within 7 days (**Table 2**). The power of daily self-reported responses to predict an MA incident injury within 7 days was below the predetermined “acceptable” level, AUC’s ranging from 0.51 to 0.66 (**Tables 1** and **2**).

**Table 1.** Association and Prediction Using the OSTRC-H Question Response for a Medical Attention Incident Injury, Within 7 days, During BMT

		Association				Prediction
Self-report questionnaire response	Variables	OR (95% CI)	*P*-value	Marginal *R*^2^	Conditional *R*^2^	Area under the curve (95% CI)
Participation	Full participation without injury/illness		Reference	0.01	0.85	0.66 (0.650-0.67)
	Full participation, but with injury/illness	2.23 (1.97-2.52)	<.01			
	Reduced participation due to injury/illness	3.19 (2.54-4.00)	<.01			
Severity	No symptoms/health problems		Reference	0.01	0.85	0.66 (0.65-0.67)
	To a mild extent	2.19 (1.91-2.51)	<.01			
	To a moderate extent	2.83 (2.38-3.36)	<.01			
	To a severe extent	4.50 (3.26-6.21)	<.01			
Location	No body region		Reference	<0.01	0.86	0.65 (0.64-0.66)
	Any-body location	2.19 (1.96-2.45)	<.01			

BMT, basic military training; OSTRC-H, Oslo Sports Trauma Research Centre Questionnaire on Health Problems.

**Table 2.** Association and Prediction Using the OSTRC-H Location Response for a Medical Attention Incident Injury to the Corresponding General Body Location, Within 7 days, During BMT

		Association				Prediction
Self-report Questionnaire Response	Variables	OR (95% CI)	*P*-value	Marginal *R*^2^	Conditional *R*^2^	Area under the curve (95% CI)
Head and neck	No head and neck complaint		Reference	<0.01	0.98	0.57 (0.43-0.71)
	Head and neck complaint	5.85 (0.44-77.43)	.18			
Spine	No spine complaint		Reference	<0.01	0.97	0.65 (0.62-0.67)
	Spine complaint	4.39 (3.07-6.30)	<.01			
Torso	No torso complaint		Reference	<0.01	0.97	0.51 (0.49-0.53)
	Torso complaint	6.34 (0.93-43.44)	.06			
Upper extremity	No upper extremity complaint		Reference	<0.01	0.97	0.63 (0.61-0.66)
	Upper extremity complaint	2.45 (1.76-3.40)	<.01			
Lower extremity	No lower extremity complaint		Reference	<0.01	0.93	0.66 (0.65-0.67)
	Lower extremity complaint	2.73 (2.40-3.11)	<.01			

BMT, basic military training; OSTRC-H, Oslo Sports Trauma Research Centre Questionnaire on Health Problems.

Measures of accuracy for daily self-reported responses are presented in **Table 3**. For “participation” and “severity” responses, where more than one response was indicative of a physical complaint, each response is presented.

**Table 3.** Accuracy for OSTRC-H Questionnaire Responses

Self-report questionnaire response	True positive (*n*)	False positive (*n*)	False negative (*n*)	True negative (*n*)	Sensitivity	Specificity	PLR	NLR	PPV	NPV	Accuracy (95% CI)
Participation	1159	6540	1156	28,026	0.501	0.811	2.646	0.616	0.151	0.960	0.791 (0.787-0.796)
*Full participation with injury/illness*	*983*	*6083*	*1156*	*28,026*	*0.460*	*0.822*	*2.577*	*0.658*	*0.139*	*0.960*	*0.800 (0.796-0.804)*
*Reduced participation due to injury/illness*	*176*	*457*	*1156*	*28,026*	*0.132*	*0.984*	*8.235*	*0.882*	*0.278*	*0.960*	*0.946 (0.943-0.948)*
Severity	1126	6678	1147	27,449	0.495	0.804	2.532	0.627	0.144	0.960	0.785 (0.781-0.789)
*To a mild extent*	*638*	*4531*	*1147*	*27,449*	*0.357*	*0.858*	*2.523*	*0.749*	*0.123*	*0.960*	*0.832 (0.828-0.836)*
*To a moderate extent*	*396*	*1853*	*1147*	*27,449*	*0.257*	*0.937*	*4.058*	*0.794*	*0.176*	*0.960*	*0.903 (0.899-0.906)*
*To a severe extent*	*92*	*294*	*1147*	*27,449*	*0.074*	*0.989*	*7.007*	*0.936*	*0.238*	*0.960*	*0.950 (0.948-0.953)*
Location	1211	7232	1240	29,627	0.494	0.804	2.518	0.629	0.143	0.960	0.785 (0.780-0.789)
Head and neck	1	518	6	38,785	0.143	0.987	10.826	0.869	0.002	1.000	0.987 (0.986-0.988)
Spine and back	90	1122	191	37,907	0.320	0.971	11.141	0.700	0.074	0.995	0.967 (0.965-0.968)
Torso	2	332	78	38,898	0.025	0.992	2.941	0.983	0.006	0.998	0.990 (0.989-0.991)
Upper extremity	102	1575	229	37,404	0.308	0.960	7.627	0.721	0.061	0.994	0.954 (0.952-0.95)
Lower extremity	826	5050	987	32,447	0.456	0.865	3.383	0.629	0.141	0.970	0.846 (0.843-0.850)

NLR,  negative likelihood ratio; NPV,  negative predictive value; OSTRC-H, Oslo Sports Trauma Research Centre Questionnaire on Health Problems; PLR,  positive likelihood ratio; PPV,  positive predictive value. For “participation” and “severity” responses, where more than one response was indicative of a physical complaint, each individual response is presented in italics.

For “participation” and “severity” responses, where more than one response was indicative of a physical complaint, each individual response is presented in italics.

## DISCUSSION

In this investigation, self-reported 12-month prior injury was not significantly associated with a greater risk of MA injury during Australian Army BMT. In contrast, daily self-reported physical complaints indicating perceived effects on “participation,” “severity,” and “location” were each significantly associated with greater odds of an MA incident injury within 7 days. Notably, the observed strength of the associations increased as perceived effects on participation and severity worsened. Additionally, spine, upper extremity, and lower extremity complaints were each independently associated with greater odds of an MA incident injury to the corresponding general body region, suggesting that it may be possible to identify location-specific injury risk during BMT. The present findings therefore provide an opportunity for secondary injury prevention strategies, focusing on early detection and intervention, to be developed within military training environments.

The current findings indicate that self-reported 12-month prior injury was not significantly associated with a greater risk of sustaining an MA injury during BMT. Hazard ratios were however relatively similar to those previously reported,^5,29^ suggesting that prior injury is related to a greater risk of MA injury during BMT. Although unclear from the present analysis, this relationship may be due to causal factors, such as inadequate rehabilitation, or due to noncausal factors, whereby prior injury history is simply a marker of other traits that result in certain individuals having a higher injury risk.^30^ Nevertheless, classifying injury history as a binary variable may not be an informative approach given the risk of subsequent injury can potentially be influenced by numerous factors such as injury severity, recovery status, time loss, and recency of the event.^31^ Indeed, injuries are complex and interactions between numerous factors can alter an individual’s injury risk over time.^9^ A one-off assessment does not therefore provide practitioners with information relating to an individual’s dynamic risk of injury.^9^

Continuous monitoring strategies may offer a more accurate assessment of injury risk.^14^ In line with previous research in sport,^18,32^ self-report responses indicative of a physical complaint were associated with greater odds of MA incident injury within 7 days. In comparison to no physical complaint, the odds of an MA incident injury within 7 days of a reported physical complaint were 2.19 to 4.50 times greater, suggesting that daily self-reported physical complaints may assist in identifying recruits at greater risk of MA injury during BMT. Although the reason for this increased risk is likely multifactorial,^9,33^ the reporting of a perceived physical complaint likely indicates the presence of pain or discomfort,^15,18^ which can gradually progress to a more serious injury.^34,35^ Notably, odds ratios increased as perceived effects on participation and severity worsened, likely due to recruits seeking MA when a physical complaint develops or begins to impact upon their ability to partake in BMT.^15^ More specifically, spine, upper extremity, and lower extremity physical complaints were each significantly associated with an MA incident injury to the corresponding general body region, suggesting that it may be possible to identify general body region injury risk during BMT. Despite having the largest odds ratios, head and neck, and torso complaints were not significantly associated with a corresponding general body region MA incident injury in 7 days. Although this finding is likely influenced by the low number of injuries to these general body regions, it should be acknowledged that recruits frequently checked these locations, indicative of an injury, while also checking “illness” (e.g., headache). These injury locations were included in the analyses; however, as no follow-up with recruits were conducted, the exact meaning of these responses (e.g., injury or illness) could not be determined and may have impacted upon results.

Associations with injury do not however imply an ability to predict individuals who will sustain an injury.^36,37^ Within the current study, using the presence of a physical complaint as an indicator of an MA incident injury within 7 days resulted in a high number of false positives (i.e., physical complaint, but no incident injury in 7 days) and false negatives (i.e., no physical complaint, but incident injury in 7 days) (**Table 3**). Consequently, the power of self-reported responses to predict an MA incident injury within 7 days was not “acceptable” (**Tables 1** and **2**). Using daily OSTRC-H responses to predict which recruits will sustain an MA injury is therefore not recommended. Positive predictive values did however increase as perceived effects on participation and severity worsened, while positive likelihood ratios greater than 1 indicate that a recruit who sustains an MA incident injury in 7 days is more likely to report a physical complaint in comparison to a recruit who does not sustain an incident injury in 7 days. Using the daily OSTRC-H as an early identification tool may therefore help identify and facilitate early intervention to help prevent minor injuries progressing to more significant ones (i.e., secondary prevention), especially considering that the majority of injuries sustained during BMT are overuse related.^2,4,5,38^ Differences in marginal (<0.01-0.01) and conditional *R*^2^ (ranging from 0.85-0.98) values do however suggest that injury management strategies should be considered at an individual level, which is unsurprising considering the diverse nature of recruit populations^39^ and subjective nature of self-reports. The reporting of a physical complaint may therefore act as a flag to facilitate communication between BMT staff and recruits.

### Limitations

When interpreting the present findings, several limitations should be considered. First, MA incident injuries may have been underestimated as data were only collected from one point of care facility (i.e., physiotherapist), and recruits may not always seek MA for an injury, or delay reporting, in an attempt to defer MA or time-loss in order to graduate from BMT on time.^40^ Furthermore, a 30-day gap rule was applied to identify incident injuries ^2,20^ and a 7-day window for an incident injury to occur within was adopted.^18^ The continued reporting of a complaint following an incident injury or reporting a complaint more than 7 days before an incident injury would therefore have been classified as a false positive. The daily reporting of physical complaints also resulted in several reports being within 7 days of each incident injury, which likely influenced the results reported. Additionally, it is acknowledged that using the OSTRC-H in isolation may be deemed a relatively reductionist approach,^9^ and differences in several other factors such as injury history, fitness levels, and workload^5,10,14^ may have impacted upon injury during BMT. However, due to large platoon sizes (up to 60 recruits), the present study aimed to evaluate the use of a simple tool to identify injury risk during BMT. Finally, while daily diaries were collected by the research team and responses had no influence upon a recruit’s training progression, recruits may have been reluctant to report physical complaints if they perceived staff may use reports to monitor recruit responses to BMT.^40^ Indeed, it has been suggested that the pain threshold to report a complaint to medical personnel during BMT is relatively high due to the potential consequences associated with injury, such as back squadding (i.e., when a recruit leaves their original training platoon and joins a platoon in an earlier week of BMT) and attrition.^15^ Consideration to the effective implementation of a daily physical complaint questionnaire in military training environments may therefore be beneficial to facilitate the early identification of physical complaints to help reduce the risk of more serious injury. However, injury burden was not considered within the present analysis; therefore, further research would be beneficial to assess if the severity of self-reported physical complaints is associated with injury burden.

## CONCLUSION

In this study, self-reported 12-month prior injury was not significantly associated with a greater risk of MA injury during BMT. Daily self-reported physical complaints indicating perceived effects on “participation,” “severity,” and “location” were each associated with greater odds of MA incident injury within 7 days, with the odds of injury increasing as perceived effects on participation and severity worsened. Furthermore, spine, upper extremity, and lower extremity physical complaints were each associated with greater odds of an MA incident injury to the corresponding general body region, suggesting that it may be possible to identify location-specific injury risk during BMT. However, due to high misclassification errors, using daily self-reported physical complaints to predict which recruits will sustain an MA incident injury in 7 days is not recommended.

## Supplementary Material

usaf185_Supplementary_Data

## Data Availability

The datasets generated and analyzed during the current study are not publicly available. Data will only be made available upon formal request to the corresponding author who will seek approval from the relevant agencies.

## INSTITUTIONAL CLEARANCE

Institutional clearance approved.
