# Supplementary material for: Associations Between Self-Reported Injury History, Physical Complaints, and Medical Attention Injury During Army Basic Military Training
Source: Mil Med. 2025 May 19;190(9-10):e2039–47. doi: 10.1093/milmed/usaf185 (PMC12459865; doi:10.1093/milmed/usaf185)
Supplement: usaf185_Supplementary_Data [file usaf185_supplementary_data.zip › Supplemetary Table 1.docx]

Table S1. Training outcome for the 625 recruits involved in the present study.

| Training Outcome | Total | Male | Female |
| --- | --- | --- | --- |
| On time | 489 | 430 | 59 |
| Pending discharge | 58 | 53 | 5 |
| Did not finish during study period | 31 | 19 | 12 |
| Delayed march out | 30 | 22 | 8 |
| APCP on time | 14 | 0 | 14 |
| APCP did not finish during study period | 2 | 0 | 2 |
| APCP delayed march out | 1 | 0 | 1 |

*APCP = Army pre-conditioning program
